# Comparative mitogenomic analyses provide evolutionary insights into the retrolateral tibial apophysis clade (Araneae: Entelegynae)

**DOI:** 10.3389/fgene.2022.974084

**Published:** 2022-09-14

**Authors:** Min Li, Min Liu, Shi-Yun Hu, Fang-Zhen Luo, Ming-Long Yuan

**Affiliations:** ^1^ State Key Laboratory of Grassland Agro-Ecosystems, Lanzhou University, Lanzhou, Gansu, China; ^2^ Key Laboratory of Grassland Livestock Industry Innovation, National Demonstration Center for Experimental Grassland Science Education, Lanzhou University, Ministry of Agriculture and Rural Affairs, Lanzhou, Gansu, China; ^3^ College of Pastoral Agricultural Science and Technology, Lanzhou University, Lanzhou, Gansu, China

**Keywords:** spiders, Entelegynae, comparative mitogenomics, evolutionary analysis, phylogeny

## Abstract

The retrolateral tibial apophysis (RTA) clade is the largest spider lineage within Araneae. To better understand the diversity and evolution, we newly determined mitogenomes of ten RTA species from six families and performed a comparative mitogenomics analysis by combining them with 40 sequenced RTA mitogenomes available on GenBank. The ten mitogenomes encoded 37 typical mitochondrial genes and included a large non-coding region (putative control region). Nucleotide composition and codon usage were well conserved within the RTA clade, whereas diversity in sequence length and structural features was observed in control region. A reversal of strand asymmetry in nucleotide composition, i.e., negative AT-skews and positive GC-skews, was observed in each RTA species, likely resulting from mitochondrial gene rearrangements. All protein-coding genes were evolving under purifying selection, except for *atp8* whose *Ka*/*Ks* was larger than 1, possibly due to positive selection or selection relaxation. Both mutation pressure and natural selection might contribute to codon usage bias of 13 protein-coding genes in the RTA lineage. Phylogenetic analyses based on mitogenomic data recovered a family-level phylogeny within the RTA; {[(Oval calamistrum clade, Dionycha), Marronoid clade], Sparassidae}. This study characterized RTA mitogenomes and provided some new insights into the phylogeny and evolution of the RTA clade.

## Introduction

The retrolateral tibial apophysis (RTA) clade (Araneomorphae: Entelegynae), composing of more than 21,500 species in 31 families, is speciose and diverse, representing nearly 45% of the known diversity of spiders. Most of the RTA clade are hunting predators and important natural enemies of many insect pests (e.g., wolf spiders, jumping spiders, running spiders, and crab spiders) (Garrison et al., 2016; Wheeler et al., 2017; Magalhaes et al., 2020). The RTA clade exhibits a worldwide distribution and is assumed to have emerged in the Jurassic period (139–161 million years ago (Ma)) (Garrison et al., 2016; Fernandez et al., 2018; Shao and Li, 2018; Magalhaes et al., 2020). According to male palp, trichobothria on the tarsi and metatarsi, the RTA clade is divided into four groups: Zodarioidea, Dionycha, Marronoid clade, and Oval calamistrum clade (Wheeler et al., 2017; Kallal et al., 2020). Araneae phylogeny has been extensively studied at various taxonomic levels by using morphological data (Coddington, 2005; Haupt, 2008), multiple molecular markers (Blackledge et al., 2009; Dimitrov et al., 2012; Wheeler et al., 2017), mitogenomic data (Pons et al., 2019; Kumar et al., 2020; Tyagi et al., 2020), and transcriptomic data (Garrison et al., 2016; Fernandez et al., 2018). These previous studies largely supported the monophyly of the RTA clade; however, the family relationships within the RTA clade remain controversial among previous studies (Wheeler et al., 2017; Fernandez et al., 2018; Kallal et al., 2020).

Typical metazoan mitochondrial genomes (mitogenomes) are circular double-stranded molecules 13–36 kb in size that usually includes 37 genes: 13 protein-coding genes (PCGs), 2 ribosomal RNA (rRNA) genes (*rrnL* and *rrnS*), and 22 transfer RNA (tRNA) genes (Cameron, 2014). In addition, mitogenomes usually have a noncoding region responsible for replication and transcription, namely the control region (CR) (Cameron, 2014). Characterized by maternal inheritance, high level of evolutionary rate, low rate of genetic recombination, and relatively conserved gene content and organization, mitogenomes have been widely used in population genetics, phylogeography, and phylogenetics (Cameron, 2014; Kumar et al., 2020; Tyagi et al., 2020). New sequencing technologies have promoted further developments of spider mitogenomes in recent years. Since the sequencing of the first spider mitogenome (*Habronattus oregonensis*) (Masta and Boore, 2004), 40 complete mitogenomes from 14 families of the RTA clade have been deposited in GenBank (as of March 2022). But relevant studies mainly focused on two characteristics exhibited by the sequenced mitogenomes of spiders, i.e., severely truncated tRNAs (Masta and Boore, 2008; Pons et al., 2019) and frequent gene rearrangements (Kumar et al., 2020; Tyagi et al., 2020; Li et al., 2021). However, other mitogenomic features of the RTA clade (e.g., nucleotide composition, codon usage, and evolutionary patterns) have not been comprehensively analyzed.

Here, we newly sequenced and annotated the mitogenomes of ten RTA species from six families, significantly increasing the number of spider mitogenomes. Combined with previously sequenced 40 RTA mitogenomes available in GenBank, we performed a detailed comparative mitogenomic analysis of the RTA clade to understand the diversity and evolution of the RTA spiders. We also reconstructed family-level relationships within the RTA clade based on three mitogenomic datasets (P123, P123RNA, and P123AA) by using two analytical methods [Bayesian inference (BI) and maximum likelihood (ML)]. This study provides some new insights into the phylogeny and evolution of the RTA clade.

## Materials and methods

### Sample collection, DNA extraction, and sequencing

Adult specimens of ten spider species from six families were collected from five counties in Qinghai Province and Gansu Province, China. Detailed information on sampling is provided in Supplementary Table S1. The collected specimens were stored in RNA later at the sampling site, then transported to the laboratory and preserved at −80°C until DNA extraction. Samples and voucher specimens were deposited in the College of Pastoral Agricultural Science and Technology, Lanzhou University in Lanzhou, China. Total genomic DNA was extracted from the spider legs of a single specimen using a Qiagen DNeasy Blood & Tissue Kit (catalog number: 69,504) according to the manufacturer’s protocols. The quality of the extracted DNA was evaluated using 1.2% agarose gel electrophoresis and spectrophotometry using a NanoDrop ND-1000 (Thermo Scientific, United States). The mitogenomes of the ten species were sequenced using Illumina NovaSeq 6,000 (2 × 150 bp) platform carried out by the Wuhan Benagen Tech Solutions Company Limited (Wuhan, China).

### Mitochondrial genome assembly and annotation

The low-quality reads (reads with a cutoff of Phred quality scores of Q20, adapter-contaminated reads, reads with more than 5% N bases, and repeated reads introduced by repeated PCR) were removed with the SOAPnuke (version: 2.1.0) (Chen et al., 2017). The high-quality reads were assembled with SPAdes (version: 3.13.0) (Dierckxsens et al., 2017), using the mitogenome of *Liphistius erawan* in GenBank as a reference.

Annotations of the assembled mitogenomes were done by using the MITOS web-server (http://mitos2.bioinf.uni-leipzig.de) (Bernt et al., 2013) with default parameters to determine the location of PCGs, tRNAs, and rRNAs. Since tRNAscan-SE was unable to detect most tRNAs of spiders, we have used other spider tRNA sequences submitted in GenBank as references to predict the boundary and length of each tRNA. All genes have been manually verified and proofread after annotation, with the principle of avoiding overlapping between genes. The tandem repeats in the CRs were predicted by the online Tandem Repeats Finder web tool (version 4.09) (https://tandem.bu.edu/trf/trf.html). All mitogenomes of ten spider species that were newly sequenced in this study have been submitted to GenBank (under accession numbers NC053648, ON419104, and ON411608–ON411615).

### Sequence analysis

Nucleotide composition and codon usage were analyzed with MEGA X (Kumar et al., 2018). Strand asymmetry was calculated using the following formulas: AT-skew = (A-T)/(A + T), GC-skew = (G-C)/(G + C) (Perna and Kocher, 1995). The effective number of codons (ENC) and the codon bias index (CBI) for each mitogenome were determined with DnaSP 5.10 (Rozas et al., 2017). The G + C contents of the 1st, 2nd, and 3rd codon positions were determined by the online CUSP tool (https://www.bioinformatics.nl/emboss-explorer/). To explain the relationship between nucleotide composition and codon bias for all the PCGs of the 50 RTA species, we analyzed the correlations between the G + C content of all codons, the G + C content of the 3rd codon positions, ENC (effective number of codons), and CBI (codon bias index). ENC-GC3 and the neutrality plot of GC12 on GC3 were generally used to determine the dominant role of either mutational forces or evolutionary forces on the codon usage bias of the genes. In this graphical plot, the regression coefficient close to zero represents a dominant role of natural selection (no effect of directional mutation pressure), while the regression coefficient close to one represents a dominant role of mutation pressure. To investigate the role of selection and better understand the evolution at the DNA level in the 50 RTA species, the value of GC percentage, synonymous substitutions per synonymous sites (*Ks*) and nonsynonymous substitutions per nonsynonymous sites (*Ka*) were also calculated using MEGA X (Kumar et al., 2018).

### Phylogenetic analysis and tree topology test

A total of 50 spider mitogenomes of 14 families, including ten newly sequenced spider mitogenomes and 40 RTA spider mitogenomes, were used for phylogenetic analyses (Supplementary Table S2). Three Liphistiidae species (*Heptathela hangzhouensis* (NC_010780), *Liphistius erawan* (NC_020323), and *Songthela* sp. (MW822557)) were used as outgroups. Protein coding genes were aligned by ClustalW (Codons) using MEGA X and translated using the invertebrate mitochondrial genetic code. The large and small ribosomal sequences were aligned by ClustalW using MEGA X. Poorly aligned and divergent sequences were removed by the Gblocks Server (http://molevol.cmima.csic.es/castresana/Gblocks_server.html). Different datasets likely contain differential phylogenetic signals and inference methods may have different potential for resolving phylogenetic relationships (Yang et al., 2015; Yuan et al., 2016; Li et al., 2022). Therefore, the alignments of PCGs and/or rRNAs were concatenated using DAMBE 5.3.74 (Xia, 2013) and three mitogenomic datasets were generated for phylogenetic analyses (Supplementary Data S1): 1) P123 dataset, with nucleotide sequences for all codon positions of 13 PCGs, comprising 9,756 nucleotides; 2) P123RNA dataset, with P123 and the nucleotide sequences of two rRNAs, comprising 10,524 nucleotides; 3) P123AA dataset, with the inferred amino acid sequences of 13 PCGs, comprising 3,252 amino acids. A test of substitution saturation for each dataset was performed with DAMBE 5.3.74 (Xia, 2013), and no dataset showed substantial substitution saturation (Supplementary Table S3). We predefined data blocks by genes and codons, e.g., 39 partitions for P123, 41 partitions for P123RNA, and 13 partitions for P123AA. The best partitioning schemes and corresponding nucleotide substitution models for each dataset were determined by using IQ-TREE 1.6.12 with default parameters (Chernomor et al., 2016), and the results were used for phylogenetic analyses (Supplementary Table S4).

ML phylogenetic analyses were conducted with software RAxML-HPC2 (Alexandros, 2014), under the GTRGAMMA model and 1,000 bootstrap replicates (BS). BI analyses were carried out using MrBayes 3.2.7 (Ronquist et al., 2012). Two independent runs with four chains (three heated and one cold) were conducted simultaneously for 1 × 10^8^ generations and each set was sampled every 100 generations. Stationarity was considered to have been reached when the estimated sample size value was over 100 and when the potential scale reduction factor approached 1.0, and the remaining parameters used default settings.

To statistically test the incongruity among tree topologies obtained from different datasets and analytical methods, a topology test was conducted with the IQ-TREE 1.6.12 (Nguyen et al., 2015). We performed SH (Shimodaira-Hasegawa test) (Shimodaira and Hasegawa, 1999), KH (Kishino-Hasegawa) (Kishino and Hasegawa, 1989), ELW (expected likelihood weight) (Strimmer and Rambaut, 2002), and AU (approximately unbiased) (Shimodaira, 2002) tests for each of the three datasets, with 1,000 replicates. These methods produce for each tree a number (*p*-value) ranging from zero to one, which represents the possibility that the tree is the true tree. The greater the *p*-value, the greater the probability that the tree is the true tree (Kishino and Hasegawa, 1989; Shimodaira and Hasegawa, 1999; Shimodaira, 2002; Strimmer and Rambaut, 2002).

## Results

### General features of the newly sequenced mitogenome

The newly sequenced mitogenomes of the ten RTA species were typical closed-circular DNA molecules, ranging from 14,171 bp in *Pardosa* sp3 to 14,755 bp in *Philodromus histrio* (Supplementary Table S2). These mitogenomes encoded 37 mitochondrial genes, i.e., 13 PCGs, 22 tRNAs, *rrnL*, and *rrnS*, among which 22 genes (nine PCGs and 13 tRNAs) were located on the majority strand (J-strand), with the remaining 15 genes on the minority strand (N-strand). A total of five gene arrangement modes were presented in the mitogenomes of 50 RTA species from 14 families (Supplementary Figure S1)*.* The most common rearrangement mode was present in 41 species of 11 families. Gene rearrangement was generally conservative within the same family, while each of the two families (Lycosidae and Salticidae) showed more than one arrangement mode. All the gene rearrangement modes in RTA mitogenomes only involved tRNAs and/or control regions (CR).

**FIGURE 1:**
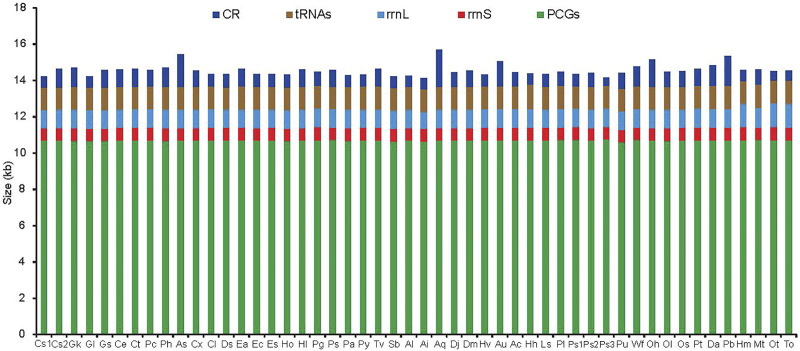
The size of PCGs, tRNAs, *rrnL*, *rrnS*, and CR among spider mitochondrial genomes of the retrolateral tibial apophysis clade. Species are abbreviated as follows: *Clubiona* sp1, CS1; *Clubiona* sp2, CS2; *Gnaphosa kompirensis*, Gk; *Gnaphosa licenti*, Gl; *Gnaphosa* sp, Gs; *Cheiracanthium erraticum*, Ce; *Cheiracanthium triviale*, Ct; *Philodromus cespitum*, Pc; *Philodromus histrio*, Ph; *Asemonea sichuanensis*, As; *Carrhotus xanthogramma*, Cx; *Cheliceroides longipalpis*, Cl; *Dendryphantes* sp, Ds; *Epeus alboguttatus*, Ea; *Evarcha coreana*, Ec; *Evarcha* sp, Es; *Habronattus oregonensis*, Ho; *Heliophanus lineiventris*, Hl; *Phanuelus gladstone*, Pg; *Phidippus* sp, Ps; *Phintella cavaleriei*, Pa; *Plexippus paykulli*, Py; *Telamonia vlijmi*, Tv; *Selenops bursarius*, Sb; *Agelena labyrinthica*, Al; *Agelena silvatica*, Ai; *Argyroneta aquatica*, Aq; *Desis jiaxiangi*, Dj; *Desis martensi*, Dm; *Heteropoda venatoria*, Hv; *Alopecosa cursor*, Au; *Alopecosa licenti*, Ac; *Halocosa hatanensis*, Hh; *Lycosa sinensis*, Ls; *Pardosa laura*, Pl; *Pardosa* sp1, Ps1; *Pardosa* sp2, Ps2; *Pardosa* sp3, Ps3; *Pirata subpiraticus*, Pu; *Wadicosa fidelis*, Wf; *Oxyopes hupingensis*, Oh; *Oxyopes licenti*, Ol; *Oxyopes sertatus*, Os; *Peucetia latikae*, Pt; *Dolomedes angustivirgatus*, Da; *Pisaura bicornis*, Pb; *Heriaeus melloteei*, Hm; *Misumenops tricuspidata*, Mt; *Oxytate striatipes*, Ot; *Thomisus onustus*, To.

The difference in mitogenome size among the 50 RTA species was primarily due to the putative CR ranging from 450 bp in *Pardosa* sp3 to 2047 bp in *Argyroneta aquatica* (Figure 1), although some of the mitogenomes have additional noncoding regions within the coding region. The largest gene overlapping regions were always located between *trnW* and *trnY*, while the largest intergenic regions were located between *trnN* and *trnA*. The newly sequenced mitogenomes of ten spider species had two genes encoding the large and small rRNA subunits (*rrnL* and *rrnS*). The former was located between *trnL1* (CUN) and *trnV*, or between *nad1* and *trnV*, while the latter was located at a conserved position between *trnV* and *trnQ*. The length of *rrnS* varied from 675 bp in *Asemonea sichuanensis* to 740 bp in *Phanuelus gladstone*, whereas the largest and smallest *rrnL* genes, were 958 bp in *Agelena silvatica* and 1,324 bp in *Oxytate striatipes* (Figure 1). The extremely truncated tRNA lengths were a typical feature of spider mitogenomes. The loss of the DHU arm and the TψC arm were common occurrences. Besides, there were many base mismatches or weak matches in the TψC arm and aminoacyl acceptor stems.

### Nucleotide composition and codon usage

**FIGURE 2:**
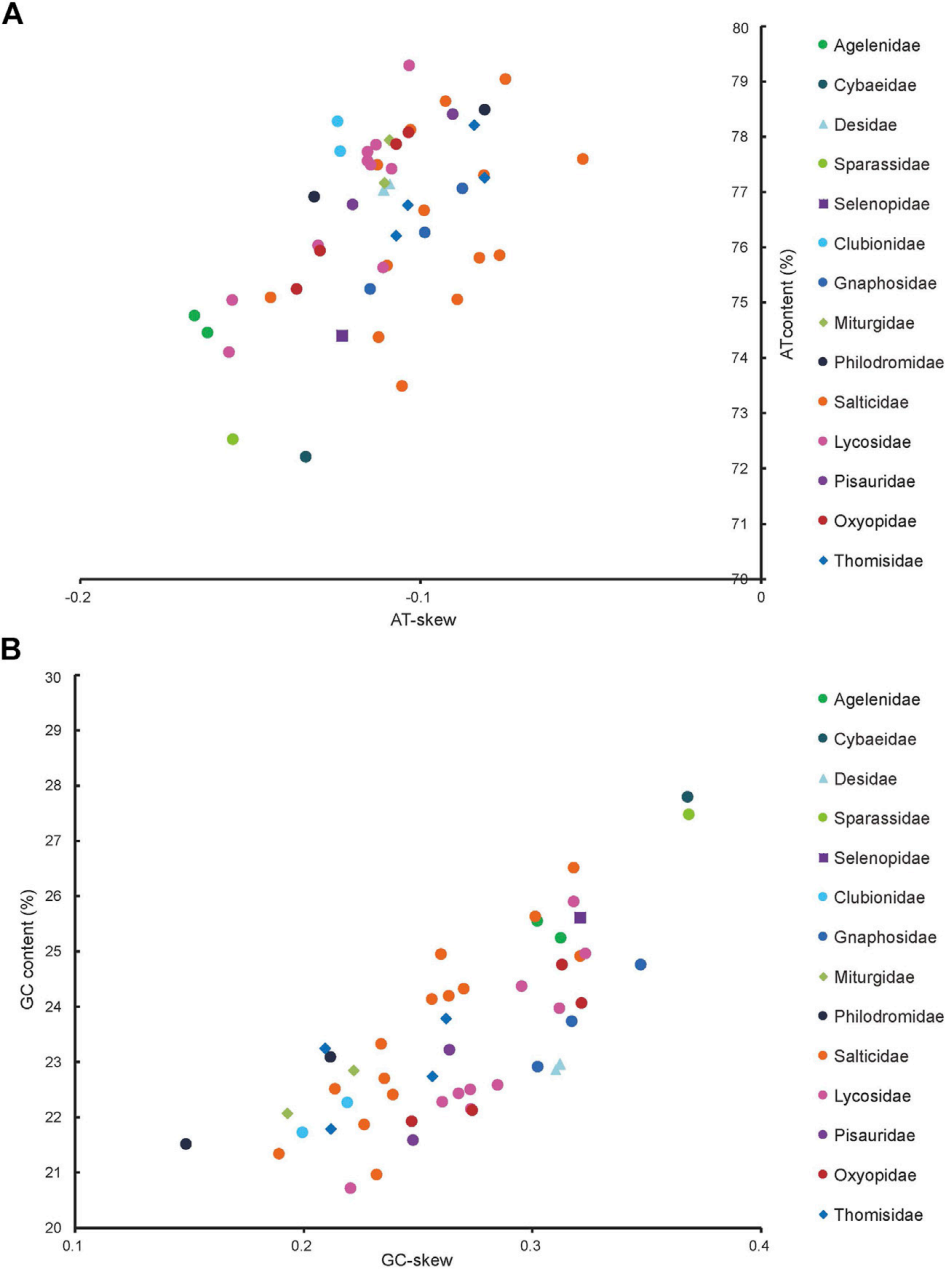
AT% vs. AT-skew and GC% vs. GC-skew in the 50 mitochondrial genomes of the retrolateral tibial apophysis clade. Measured in bp percentage (*Y*-axis) and level of nucleotide skew (*X*-axis). Values are calculated on J-strands for full-length mitochondrial genomes. **(A)** A + T% vs. AT-skew; **(B)** G + C% vs. GC-skew. See Figure 1 for the full names of the species.

**FIGURE 3:**
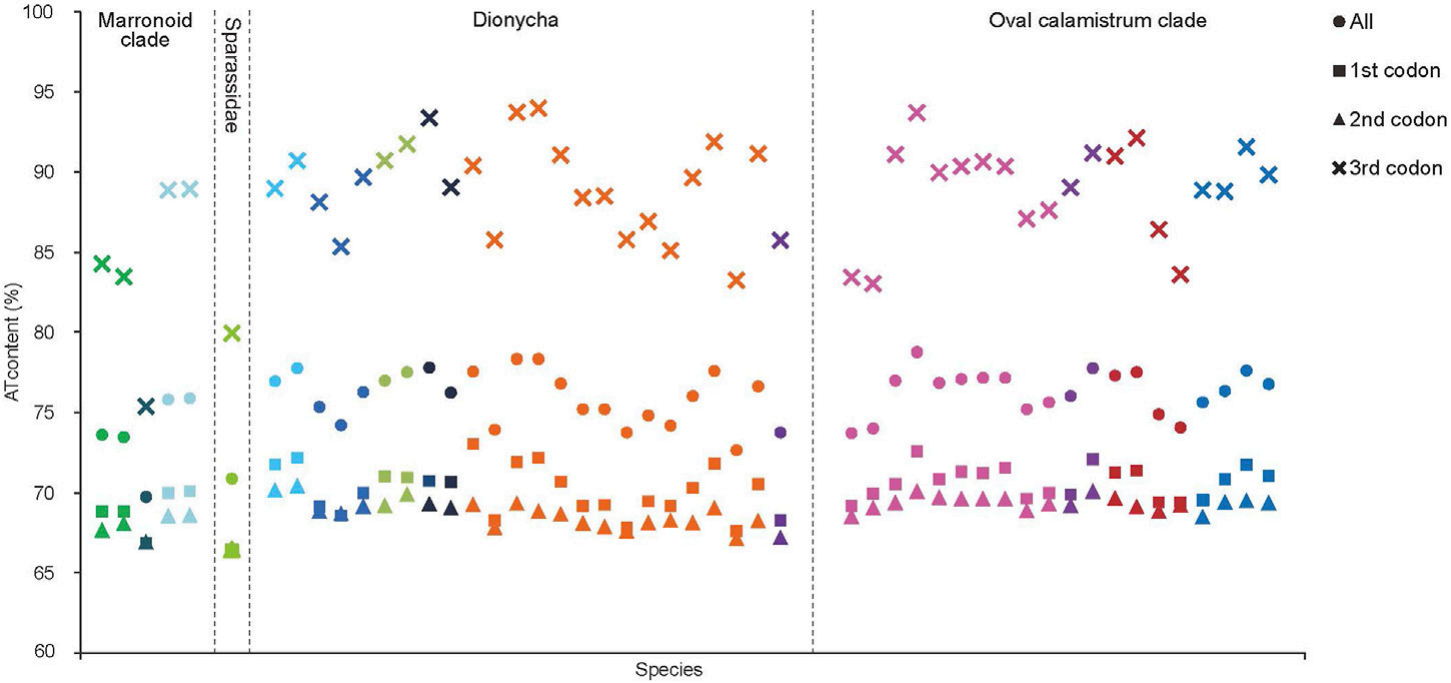
A + T% of the mitochondrial protein-coding genes among three groups within the retrolateral tibial apophysis clade. The colors of the symbols match those in Figure 2.

The J-strand of the newly sequenced mitogenomes of the ten RTA species were biased toward A and T (Figure 2). The 50 RTA mitogenomes presented a similar nucleotide composition, that is, high A + T content (77.3–79.2%), negative AT-skews (−0.166∼ −0.052), and positive GC-skews (0.312–0.239) (Figure 2). A + T content varied for each codon position of the concatenated 13 PCGs for the 50 RTA spiders: the third codon position had a higher A + T content (75.4–94.0%) than that of the first (66.4–73.0%) and second (66.3–70.4%) positions (Figure 3). Different codon numbers were found in the 50 RTA spider species, ranging from 3,532 in *Pirata subpiraticus* to 3,583 in *Pardosa* sp3 (Supplementary Table S5). The results of the relative synonymous codon usage (RSCU) analysis reflected that 62 mitochondrial codons of invertebrates were used in the mitogenomes of 20 RTA spiders, while 61, 60 and 59 mitochondrial codons were used in the mitogenomes of 15 species, 13 species and two species (*Pisaura bicornis* and *Alopecosa cursor*), respectively (Supplementary Table S5). Fifty-three codons were used in all the mitogenomes of RTA species, while five GC-rich codons CCG (P), GCG (A), CGC (R), CGG (R), and GGC (G) were not used in at least one species. Comparative analysis indicated that codon usage modes and major customarily utilized codons of the 50 mitogenomes of RTA spiders were highly conserved. Four AT-rich codons [UUA (L), UUU (F), AUU (I), and AUA (M)] were the most frequently used in all the RTA species (Supplementary Table S5). The RSCU values of the PCGs revealed that there was a higher frequency in the usage of AT than that of GC in the third codon positions.

**FIGURE 4:**
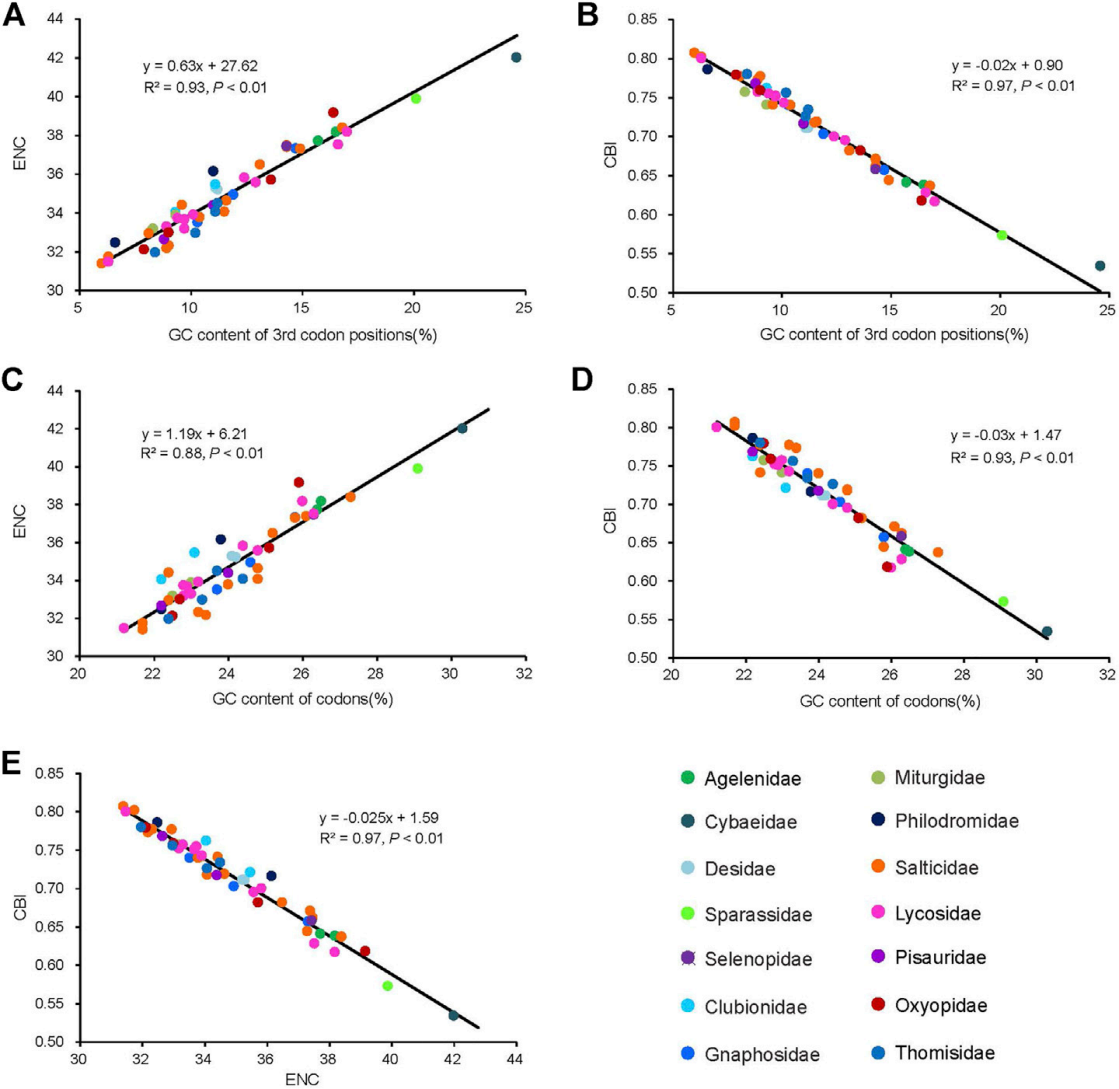
Evaluation of codon bias in the mitochondrial genomes of 50 spider species of the retrolateral tibial apophysis clade. G + C%, G + C content of all codon positions; ENC, effective number of codons; CBI, codon bias index. The colors of the symbols match those in Figure 2.

**FIGURE 5:**
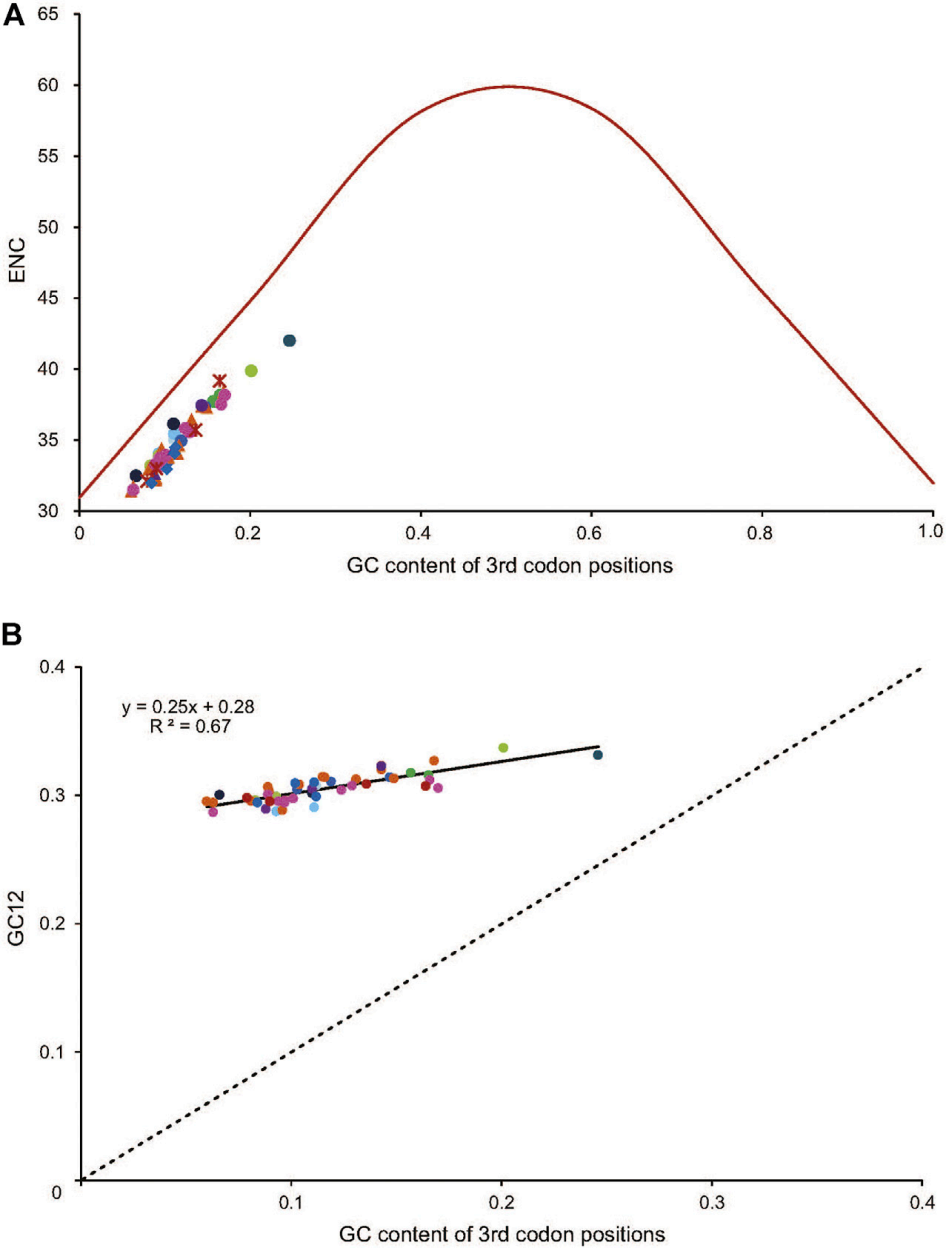
The correlation between the effective number of codons (ENC) and G + C content of the third codon positions (GC3) for 50 spider species of the retrolateral tibial apophysis clade. The colored dots match those in Figure 2. **(A)** The solid line represents the relationship between the ENC* (2 + GC3 + (29/[(GC3)^^2^ + (1-GC3)^^2^]) and the GC3 content. **(B)** The solid line represents the relationship between the ENC and the GC3 content, whereas the dotted line indicates y = x. GC12, G + C content of the first and second positions.

The average of the ENC values for all the PCGs was 34.95, ranging from 31.40 (*Dendryphantes* sp.) to 42.00 (*Argyroneta aquatica*). A positive correlation was found between ENC and G + C content for all codons (*R* ^2^ = 0.97, *p* < 0.01) (Figure 4A) and the 3rd codon positions (*R* ^2^ = 0.99, *p* < 0.01) (Figure 4B). Furthermore, a negative correlation was found between CBI and G + C content for all codons (*R* ^2^ = 0.96, *p* < 0.01) (Figure 4C), G + C content of the 3rd codon positions (*R* ^2^ = 0.97, *p* < 0.01) (Figure 4D) and ENC (*R* ^2^ = 0.98, *p* < 0.01) (Figure 4E). To further investigate the codon usage bias, ENC and the G + C content were analyzed for the 13 PCGs of all the 50 RTA mitogenomes. A neutrality plot of GC12 versus GC3 analysis was performed to determine the extent of mutation and selection forces in the codon usage bias among the genes. The actual ENC values for all RTA spiders were just below the ENC curve indicating that codon bias might mainly be influenced by natural selection (Figure 5A). The GC12 and GC3 plot showed a significant positive correlation between GC12 and GC3 (*R* ^2^ = 0.82, *p* < 0.01) (Figure 5B), and the regression coefficient of GC12 on GC3 was 0.25.

### Long non-coding region

**FIGURE 6:**
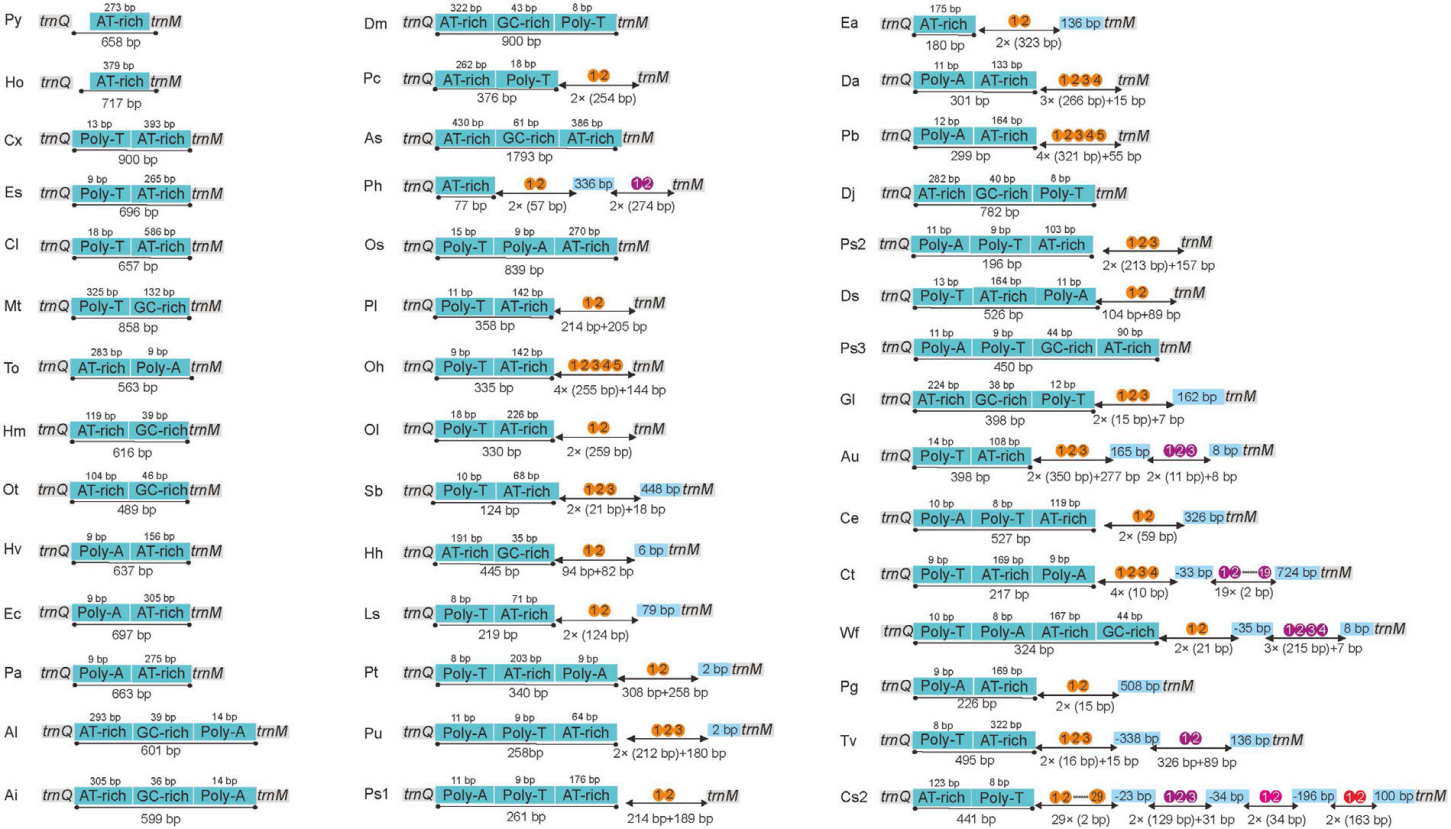
Organization of the control regions in the RTA mitochondrial genomes. The location and copy number of tandem repeats are shown in orange and three different depths of red with arabic numbers inside. The boxes colored sky blue represent interval sequences (positive numbers) or overlaps (negative numbers) between two elements.

The CRs (which were A + T-rich regions) in the 50 RTA mitogenomes were always located between *trnQ* and *trnM* (Supplementary Figure S1). Our results showed that all the RTA species had only one CR. The size of CRs was highly variable among the 43 complete mitogenomes, ranging from 450 (*Pardosa* sp3) to 1793 bp (*Asemonea sichuanensis*). All the complete RTA mitochondrial CRs are A + T-rich, composed of more than 65.6% of A + T. A comparison of structures in CRs among RTA mitogenomes is shown in Figure 6. Some essential elements were observed among the RTA mitogenomes: 1) AT-rich region; 2) GC-rich region; 3) Poly-T; 4) Poly-A. Besides, there are many TATA motifs and G(A)nT motifs. Large tandem repeat units were also found in CRs of 24 RTA mitogenomes. There were four tandem repeat units in the CR of *Clubiona* sp2, and the other 23 species contained one or two tandem repeat units. There were large differences in the copy number of each tandem repeat unit, ranging from two in 12 species to 29 in *Clubiona* sp2. However, large tandem repeat units were not discovered in some RTA mitogenomes: *Pardosa* sp3, *Oxyopes sertatus,* eight species of the family Salticidae, and all species of family Agelenidae, Desidae, and Thomisidae.

### Evolutionary rates

**FIGURE 7:**
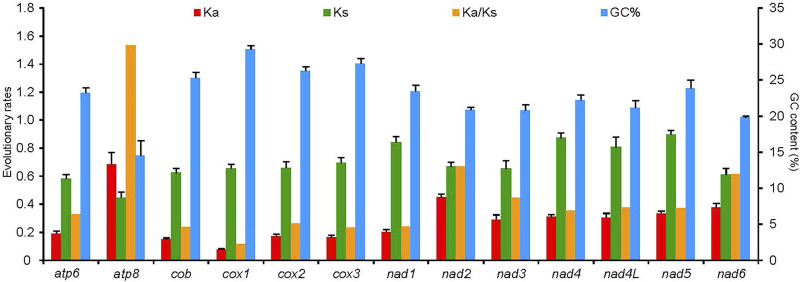
Evolutionary rates of 13 protein-coding genes in the mitochondrial genomes of 50 spider species of the retrolateral tibial apophysis clade. The left *Y*-axis provides the substitution rate of the mitochondrial gene, while the right *Y*-axis provides the G + C content. Synonymous nucleotide substitutions per synonymous site (*Ks*) and nonsynonymous nucleotide substitutions per nonsynonymous site (*Ka*) are calculated using the Kumar method. The standard error estimates are obtained by a bootstrap procedure (1,000 replicates).

The average *Ks* and *Ka* values of the 50 RTA species were different among 13 PCGs, and all values were less than 1 (Figure 7). Conversely, *Ka* showed a great variation, of which *atp8* (0.69) was the largest, which indicated that *atp8* had the fastest evolutionary rate. Moreover, *nad2* and *nad6* also presented a faster evolutionary rate, while *cox1* showed the slowest evolutionary rate. *Ka*/*Ks* values of all PCGs were less than 1 except *cox1*. *Ka*/*Ks* values of the 13 PCGs were negatively correlated with the GC percentage (*R* ^*2*^ = 0.76, *p* < 0.01).

### Mitochondrial phylogeny of the RTA clade

**FIGURE 8:**
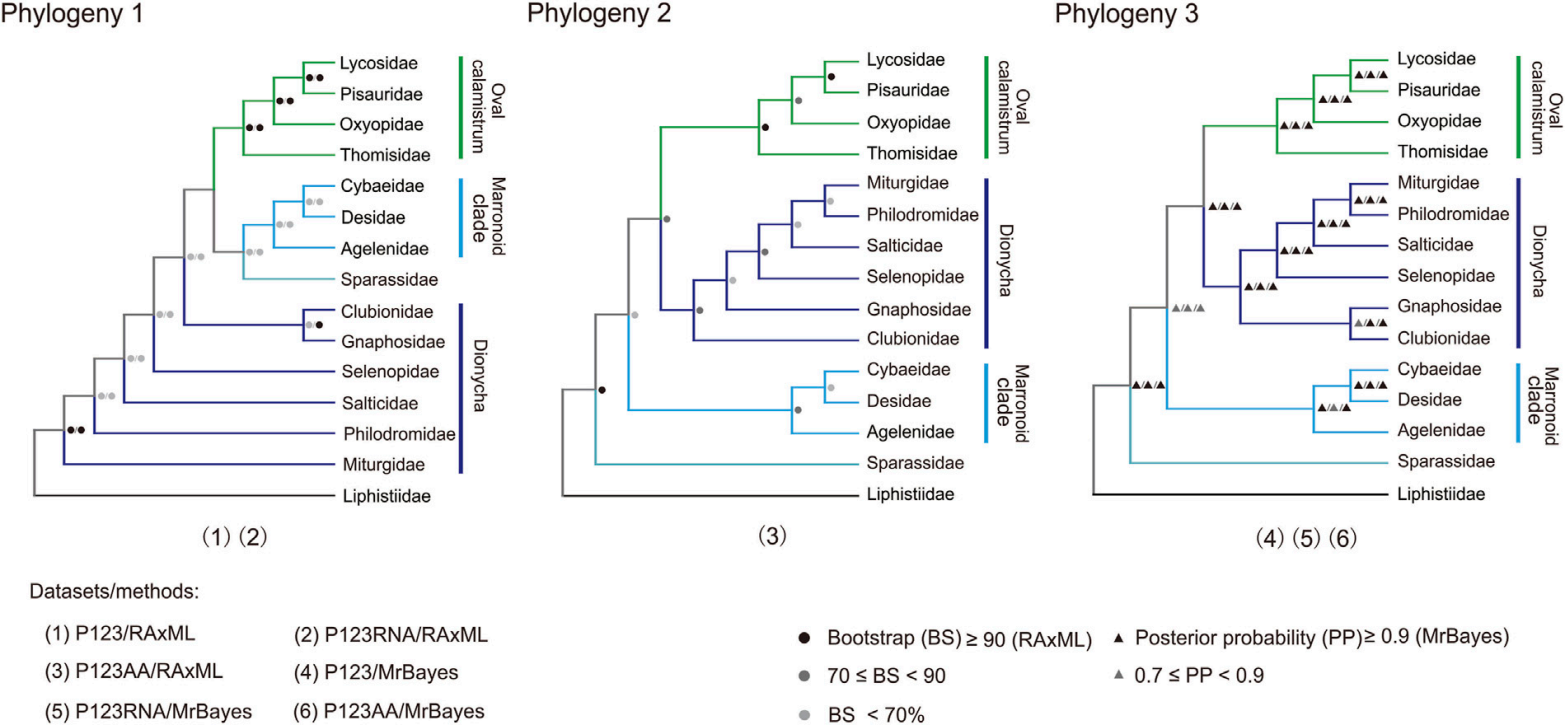
Three phylogenies of 14 families in the retrolateral tibial apophysis clade. Phylogeny 1 is summarized: P123 and P123RNA datasets based on RAxML analysis); Phylogeny 2 from P123AA dataset based on RAxML analysis; Phylogeny 3 from three datasets based on MrBayes analysis. P123, with nucleotide sequences for all codon positions of 13 PCGs; P123RNA, with P123 and the nucleotide sequences of two rRNAs; P123AA, with the inferred amino acid sequences of 13 PCGs. Icons on branches are bootstrap values and Bayesian posterior probabilities. Numbers 1–6 indicated phylogenetic relationships among the 14 families in the RTA clade obtained with different mitogenomic datasets and analytical methods.

Three tree topologies were obtained using three mitogenomic datasets and two analytical methods (BI and ML) (Figure 8; Supplementary Figure S2; Supplementary Data S2), totally involving 50 RTA species of 14 families (Supplementary Table S2). The two phylogenetic trees constructed with two datasets (P123 and P123RNA) through ML method gained the same relationship between families (Phylogeny 1 in Figure 8; Supplementary Figures S2A,B). The three phylogenetic trees constructed with three datasets through the BI method also gained the same relationship between families (Phylogeny 3 in Figure 8; Supplementary Figures S2D–F). Tree topology tests indicated that the topology summarized from phylogenetic trees based on three mitogenomic datasets using the BI method represented the most likely topology (Supplementary Table S6; Phylogeny 3 in Figure 8; Supplementary Figures S2D–F).

All the six phylogenetic trees based on mitogenomic data covered the monophyly of Oval calamistrum clade, Marronoid clade, and each family containing more than two species (most BS values >70, all posterior probability (PP) values ≥0.99) except Pisauridae (Figure 8; Supplementary Figure S2). The family Pisauridae was non-monophyletic in three phylogenetic trees (Supplementary Figures S2A,C,F), but it was monophyletic in the other three (Supplementary Figures S2B,D,F). The monophyly of Dionycha was covered by the ML tree using the P123AA dataset (BS > 70, Figure 8) and BI trees using three datasets (PP > 0.9, Figure 8). The most likely RTA phylogeny, based on the tree topology test results (Supplementary Table S6), supported (((Oval calamistrum clade, Dionycha), Marronoid clade), Sparassidae). All analyses supported a close relationship between Cybaeidae and Desidae within the Marronoid clade (BS < 70, PP > 0.9; Figure 8). Within the Oval calamistrum clade, Oxyopidae was supported as the sister to Lycosidae plus Pisauridae, along with Thomisidae at the basal position (PP > 0.9, Figure 8).

## Discussion

### Mitochondrial genome organization and composition

The newly sequenced mitogenomes of the ten RTA species contained the typical gene contents that have been hypothesized for other spiders (Pons et al., 2019; Zhu et al., 2019; Tyagi et al., 2020). These ten RTA mitogenomes were biased toward A and T, consistent with previous findings (Zhu et al., 2019; Li et al., 2020). Arthropod mitogenomes tend to have a positive AT-skew and a negative GC-skew on the J-strand, i.e., most species showed the strand asymmetry (Wang et al., 2017; Ye et al., 2021). However, the strength of the skews in the mitogenomes of the RTA clade showed the opposite pattern, indicating that the J-strands of these mitogenomes have strand asymmetry reversal and favored T and G. This seemed to be a common phenomenon in Araneae (Wang et al., 2016). And similar modes of nucleotide skew were also found in mitogenomes of some arthropod taxa, such as those of scorpions (Masta et al., 2009), insects (Monnens et al., 2020; Yan et al., 2021) and other arthropods (Zapelloni et al., 2021). Through comparison of the nucleotide skews among all the sequenced spider mitogenomes, the divergence between the two suborders was detected (Wang et al., 2016). All the Opisthothelae spider mitogenomes showed a negative AT-skew but a positive GC-skew, while mitogenomes of two species from Mesothelae were characterized by a positive AT-skew and negative GC-skew. These results indicated that a reversal bias in nucleotide composition (the generally AC-rich J-strand turned to be unusually GT rich) arose after the divergence of Opisthothelae spiders from their common ancestor with Mesothelae spiders, and since then this trait was retained in the evolutionary process of Opisthothelae spiders (Zhu et al., 2019). The opposite directions of GC-skews of J-strand in *H. hangzhouensis* and RTA mitogenomes may suggest that the N-strand was replicated first in *H. hangzhouensis* and that the J-strand was replicated first in RTA mitogenomes. Because the parental strand was displaced by its daughter strand and remained single-stranded until the lagging strand was synthesized, the strand displaced earlier would presumably be more susceptible to hydrolytic deamination (Yang et al., 2005).

Mitochondrial gene order is usually highly conserved in Arthropod mitogenomes (Cameron, 2014). The 50 RTA mitogenomes shared five kinds of gene arrangement modes and were not consistent with the ancestor *Limulus polyphemus* (Kumar et al., 2020; Tyagi et al., 2020). Previous studies have been conducted to explore the relationship between gene arrangement and reversal of strand asymmetry in insect mitogenomes, indicating that all species that exhibited strand asymmetry reversal in the mitogenomes were found to have accelerated rates of gene rearrangement, whereas species with accelerated gene rearrangements did not always exhibit strand asymmetry reversal (Cameron et al., 2007; Wei et al., 2010; Li et al., 2012). The results indicated an inversion of CR led to a reversal of chain asymmetry and the sign of GC-skew was associated with replication orientation when the sign of AT-skew varies with gene direction, replication and codon position (Wei et al., 2010). All RTA mitogenomes exhibited gene rearrangements and a reversal of strand asymmetry, and it is guessed that the reversal of strand asymmetry on the J-strand of the 50 RTA mitogenomes may be caused by gene rearrangement.

### Codon usage bias and mutations

The synonymous codons are used with different frequencies in the coding regions of the mitogenomes, which leads to codon usage bias (Wei et al., 2014). Codon usage bias is a fundamental phenomenon and an important evolution event that has been reported in many organisms (Wei et al., 2014; Abdoli et al., 2022). Some hypotheses have been proposed for the cause of codon usage bias, and the main influencing factors for codon usage are the mutation pressure and natural selection. The higher frequency in the usage of AT than that of GC in the third codon positions has been observed in the RTA mitogenomes, as it was also found in non-RTA species (Li et al., 2020; Zhang et al., 2021) and insects (Abdoli et al., 2022). The average of the ENC values for all the PCGs of the RTA species was less than 35, which indicated a strong codon bias (Sharp et al., 1993).

It is widely accepted that there was no contribution of selection force when a change occurred in the third codon position of a synonymous codon as the corresponding amino acid remains the same. In our analysis, the ENC and GC3 plot indicated that codon bias might mainly be influenced by natural selection (e.g., generation time, body size, and habitat), as has been found in non-RTA species (Kumar et al., 2020). Besides, the significant positive correlation between GC12 and GC3 indicated mutational forces (GC mutation bias) might be involved in leading to the codon bias in RTA mitogenomes. The regression coefficient of GC12 on GC3 was less than 0.5, indicating natural selection might have played a major role in shaping codon usage bias, while mutation pressure might have played a minor role in shaping codon usage bias. It has been reported that codon usage bias can be triggered by several other factors such as the content of nucleotides, gene length, gene expression level, protein secondary structure, the evolutionary age of genes in metazoan genomes, and the external environment (Héctor et al., 2000; Uddin and Chakraborty, 2019).

### Long non-coding region

The long non-coding regions of the mitogenomes were defined as the CRs according to other spider mitogenomes (Tyagi et al., 2020; Li et al., 2021). The CR of animal mitogenomes played a vital role in the initiation of transcription and gene replication process (Saito et al., 2005; Ki et al., 2010). All the RTA mitogenomes only contained one CR which was conserved at their position between *trnQ* and *trnM*, consistent with most spider mitogenomes (Pons et al., 2019; Tyagi et al., 2020), indicating that the number and location of CR were relatively conserved in Araneae.

Some conserved elements (i.e., AT-rich region, G-C rich region, TATA motif, Poly-T stretch, and G(A)nT motif) were found in the CRs of the RTA mitogenomes, as has been found in non-RTA mitogenomes (Kumar et al., 2020; Tyagi et al., 2020). These important elements have been identified as initiation sites for gene replication and transcription (Cameron et al., 2007). The CRs of arthropods were often divided into four parts: Poly-T stretch, tandem repeat sequences, an A + T rich subregion, and stem-loop structures (Cook, 2005; Zhao et al., 2011). However, tandem repeat sequences were not found in the CRs of some RTA species and the CRs could not be divided into distinct conserved or variable domains, which indicated the diversity of CR structures in the mitogenomes of the RTA clade. The fact that tandem repeat sequences were not conserved among these RTA mitogenomes may lead to the size variation of CRs and indicate a lack of a functional role of the tandem repeat sequences (Li et al., 2012). In contrast to tandem repeat sequences, stem-loop structures were generally found in RTA CRs, as has been shown in previous studies which involved non-RTA species (Kumar et al., 2020; Tyagi et al., 2020). The stem-loop structures have been reported to function as splicing recognition sites during the process of the transcripts (He et al., 2005). Therefore, the stem-loop structures of these RTA CRs may also play an important role in the process of gene replication and transcription.

### Evolutionary rates

Estimating the *Ka*/*Ks* of PCGs is considered a useful method to quantify the effects of natural selection on adaptive evolution (Ohta, 1992). It is widely accepted that *Ka*/*Ks* > 1*, Ka* = Ks, and *Ka*/*Ks* < 1 generally represented positive selection, neutral mutation, and purifying selection, respectively (Meiklejohn et al., 2007). The *Ka*/*Ks* values for 12 PCGs of all the RTA mitogenomes were far lower than 1, which was seen to be the case in most of the arthropod mitogenomes, i.e., spiders (Li et al., 2021), spider mites (Sun et al., 2018) and insects (Wang et al., 2017; Cao et al., 2019; Chang et al., 2020), suggesting that these genes were evolving under purifying selection. These low *Ka*/*Ks* values indicated that purifying selection generally dominated mitogenome evolution of the RTA clade, which may be due to its importance in cellular respiration (Meiklejohn et al., 2007). It was also reported that purifying selection could eliminate harmful mutations in the organisms and predominate the evolution of mitogenomes (García and Oteo, 2019).

The *atp8* gene had the highest *Ka*/*Ks* value, consistent with the previously identified *Ka*/*Ks* ratios in spider species (Kumar et al., 2020; Li et al., 2021), and insects (Chakraborty et al., 2018; Li et al., 2019; Chang et al., 2020; Ye et al., 2021). It has been reported that different mitochondrial PCGs evolved at different rates (Li et al., 2012; Zapelloni et al., 2021), as has been found in RTA species. The *Ka*/*Ks* ratios of *atp8* >1 indicated that *atp8* was under strong pressure of positive selection and might evolve more quickly than the other 12 PCGs in the spider mitogenomes (Chen et al., 2021; Li et al., 2021). It was reported that positive selection may help organisms adapt to their environment (Rasmus, 2005; Sun et al., 2018). Thirteen PCG genes in the arthropod mitogenomes, as well as some nuclear genes encoding protein subunits, together constitute four of the five complexes in the electron transport chain (ETC) of the oxidative phosphorylation (OXPHOS) pathway (Finch et al., 2014). The *atp8* gene was a core subunit of the Complex V (ATP synthase), which played an important role in phosphorylating ADP to synthesize an adenosine triphosphate molecule (Finch et al., 2014). Positive selection was found in genes involved in the OXPHOS pathway, i.e., *atp6* (Li et al., 2018; Yuan et al., 2020; Zapelloni et al., 2021), *nad5* (Li et al., 2018; Yuan et al., 2020; Zapelloni et al., 2021), and *atp8* (Li et al., 2018; Zapelloni et al., 2021), suggesting that the evolution of energy metabolism genes was critical for adaptation to new environments (Castoe et al., 2008; Castoe et al., 2013). The *atp8* gene in RTA mitogenomes may have experienced a positive selection in adaption to the environment. The *cox1* had the lowest *Ka*/*Ks* ratio, as has been found in most of the arthropod mitogenomes (Chakraborty et al., 2018; Li et al., 2019; Ye et al., 2021), which represented fewer changes happened in amino acids and this gene was the most conserved one (Tyagi et al., 2017). Hence *cox1* is widely used as a potential molecular marker for species identification and phylogenetic analysis. Correlation between *Ka*/*Ks* values of the 13 PCGs and the GC content indicated that the variation of GC content may have caused different evolution patterns of PCG genes.

### Phylogeny of the RTA clade

Mitogenomic data have been widely used to explore phylogenetic relationships among different arthropod groups, e.g., spiders (Kumar et al., 2020; Tyagi et al., 2020; Li et al., 2021), Acari (Arribas et al., 2019) and insects (Haran et al., 2013; Zhang et al., 2021). The phylogenomic analyses presented in this paper represent the largest assessment of RTA phylogeny using mitogenome data in terms of the number of spider species. Consistent with the results of previous studies (Kallal et al., 2020; Kulkarni et al., 2021), all the six phylogenetic trees based on mitogenomic data covered the monophyly of two groups (Oval calamistrum clade and Marronoid clade) and families with more than two species excluding Pisauridae. The non-monophyletic Pisauridae has also been reported paraphyletic in previous phylogeny analyses (Garrison et al., 2016; Wheeler et al., 2017). The monophyly of Oxyopidae and Gnaphosidae was always covered in our study. It was worth noting that these three Gnaphosidae species were from the same genus, thus the monophyly of Gnaphosidae needs to be further examined using species from different genera. Polotow et al. (2015) found a monophyletic Oxyopidae (Polotow et al., 2015), but in the analysis based on six molecular markers, *Senoculus* (Araneae: Senoculidae) was allied to some Oxyopidae species, leading to diphyletic Oxyopidae (Wheeler et al., 2017).

The most likely RTA phylogeny supported a phylogeny of {[(Oval calamistrum clade + Dionycha) + Marronoid clade]+ Sparassidae}, which was a little different from previous studies in which a sister relationship between Marronoid clade and Sparassidae was supported (Kallal et al., 2020; Kulkarni et al., 2021). All the analyses highly supported a close relationship between Desidae and Cybaeidae, this result was consistent with previous studies based on mitogenome (Tyagi et al., 2020; Li et al., 2021) and transcriptome data (Garrison et al., 2016; Kulkarni et al., 2021). The systematic position of the species *Argyroneta aquatica* under family Cybaeidae or Dictynidae was still controversial (Tyagi et al., 2020). In addition, which family the genus *Cheiracanthium* belonged to was also under discussion (Wheeler et al., 2017; Tyagi et al., 2020). Our analyses supported Oxyopidae as the sister to Lycosidae plus Pisauridae, along with Thomisidae at the base position of the Oval calamistrum clade, which was consistent with recent studies (Garrison et al., 2016; Kulkarni et al., 2021; Li et al., 2021), but different with the results of Fernandez et al. (2018) in which Thomisidae gathered with Oxyopidae. Within Dionycha, the most likely topology recovered in this study showed that Gnaphosidae grouped with Clubionidae, whereas the remaining four families (i.e., Miturgidae, Selenopidae, Philodromidae, and Salticidae) clustered together. However, the internal relationships among them were unstable, as reported by a large molecular phylogenetic study (Wheeler et al., 2017). Notably, due to a single sequenced mitogenome in several families, such as Selenopidae and Cybaeidae, it likely limited the achievement of more phylogenetic information. Thus, to obtain more clarity, it is important to sequence the mitogenomes of more families and more species in each family. It is recommended that nuclear genes, a larger number of samples, and a combination of mitogenome data and morphological data, should be considered in the future to study the phylogeny of the RTA clade more accurately.

Although both mitogenomic datasets and analytical methods exert important impacts on tree topologies, mitogenomic data have important implications in phylogenetic studies on the RTA clade. The present study shows that mitogenomes are a useful tool for providing a robust phylogeny of spiders, even though the position of certain families could not be well determined. However, this problem also applies to other molecular data (Wheeler et al., 2017; Fernandez et al., 2018; Kallal et al., 2020). The family diversity in this study still only represents less than half of the total family count of the RTA clade. Therefore, further research is needed that combines molecular (i.e., transcriptome, genome, and mitogenome) and morphological data under denser taxon sampling to explore the phylogenetic relationship of the RTA clade.

## Conclusion

In this study, we sequenced and annotated the complete or nearly complete mitogenomes of ten spider species, which further enriched the number of mitogenomes of Araneae. The comparative mitogenomic analyses showed that RTA mitogenomes are highly conserved in gene contents, base composition, and codon usage. Phylogenetic relationships within RTA based on mitogenomic data are similar with the previous molecular classification, suggesting that mitogenomic data could provide much useful information for resolving the RTA phylogeny. This study was valuable for further understanding of phylogenetic relationships among families within the RTA clade. Sequencing more mitogenomes representing various taxonomic levels, particularly from closely related species, will not only improve the accuracy of annotations for spider mitochondrial genes, but will also greatly improve our understanding of mitogenomic evolution and phylogenetic relationships in spiders.

## Data Availability

The data presented in the study are deposited in the GenBank repository, accession numbers NC053648, ON419104, and ON411608–ON411615.

## Supplementary material

Supplementary Figure S1 Gene rearrangements in the mitochondrial genomes of 50 spider species of the retrolateral tibial apophysis clade. Genes on the minor strand are indicated with arrows oriented to the left, while genes located on the major strand are indicated with arrows oriented to the right. Different types of genes are marked with different color backgrounds: tRNAs use bright yellow and blue, protein-coding genes (PCGs) use pink and green, ribosomal genes (rRNAs) use bright green, and control regions are marked in red. PCGs and rRNAs are shown with standard abbreviations. Transfer RNA genes are abbreviated by the one-letter amino acid code, with L1 = CUN, L2 = UUR, S1 = AGN, and S2 = UCN.

Supplementary Figure S2 Phylogenetic relationships among 14 families within the retrolateral tibial apophysis clade were obtained from three datasets (P123 dataset, P123AA dataset, and P123RNA dataset) and two analytical methods (maximum likelihood method and Bayesian inference using RAxML and MrBayes, respectively).
