# Supplementary material for: Comparative mitogenomic analyses provide evolutionary insights into the retrolateral tibial apophysis clade (Araneae: Entelegynae)
Source: Front Genet. 2022 Sep 14;13:974084. doi: 10.3389/fgene.2022.974084 (PMC9515440; doi:10.3389/fgene.2022.974084)
Supplement: Supplementary file 1 [file DataSheet1.ZIP › Additional files/Figure S1 Gene arrangements of RTA.pdf]

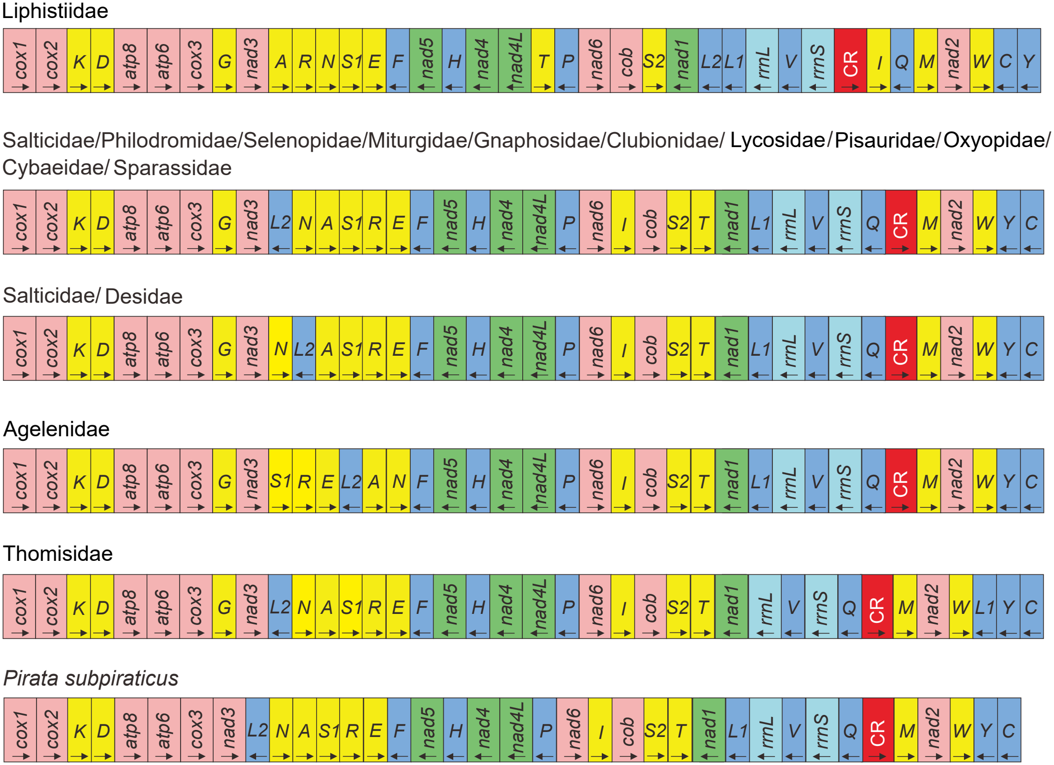
Liphistiidae
cox1 cox2 K D atp8 atp6 cox3 G nad3 A R N S1 E F nad5 H nad4 nad4L T P nad6 cob S2 nad1 L2 L1 rrnL V rrnS CR I Q M nad2 W C Y
Salticidae/Philodromidae/Selenopidae/Miturgidae/Gnaphosidae/Clubionidae/ Lycosidae/ Pisauridae/ Oxyopidae/ Cybaeidae/ Sparassidae
cox1 cox2 K D atp8 atp6 cox3 G nad3 L2 N A S1 R E F nad5 H nad4 nad4L P nad6 I cob S2 T nad1 L1 rrnL V rrnS Q CR M nad2 W Y C
Salticidae/ Desidae
cox1 cox2 K D atp8 atp6 cox3 G nad3 N L2 A S1 R E F nad5 H nad4 nad4L P nad6 I cob S2 T nad1 L1 rrnL V rrnS Q CR M nad2 W Y C
Agelenidae
cox1 cox2 K D atp8 atp6 cox3 G nad3 S1 R E L2 A N F nad5 H nad4 nad4L P nad6 I cob S2 T nad1 L1 rrnL V rrnS Q CR M nad2 W Y C
Thomisidae
cox1 cox2 K D atp8 atp6 cox3 G nad3 L2 N A S1 R E F nad5 H nad4 nad4L P nad6 I cob S2 T nad1 rrnL V rrnS Q CR M nad2 W L1 Y C
Pirata subpiraticus
cox1 cox2 K D atp8 atp6 cox3 nad3 L2 N A S1 R E F nad5 H nad4 nad4L P nad6 I cob S2 T nad1 L1 rrnL V rrnS Q CR M nad2 W Y C
