# Supplementary figures and images for: Comparative mitogenomic analyses provide evolutionary insights into the retrolateral tibial apophysis clade (Araneae: Entelegynae)
Source: Front Genet. 2022 Sep 14;13:974084. doi: 10.3389/fgene.2022.974084 (PMC9515440; doi:10.3389/fgene.2022.974084)

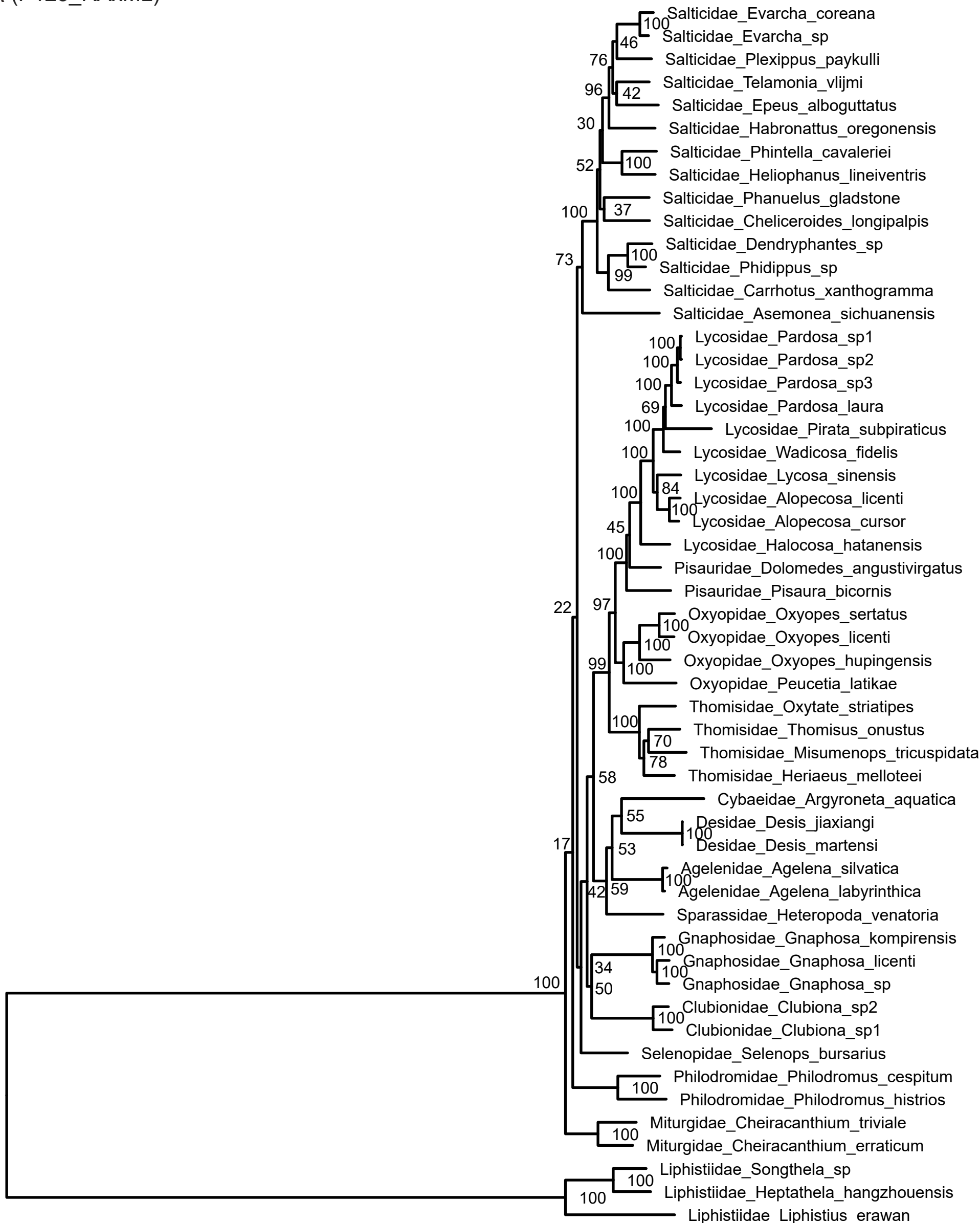

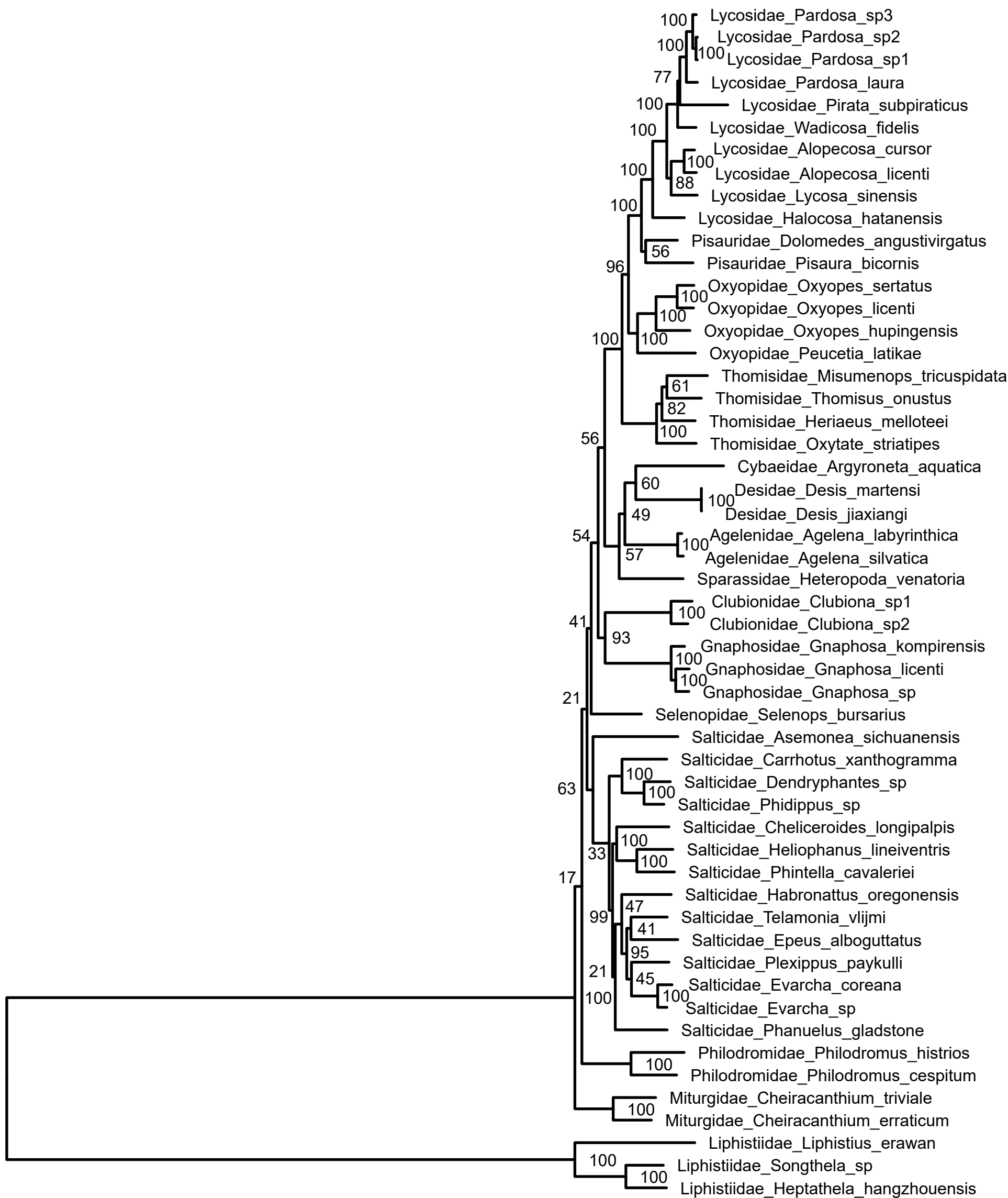

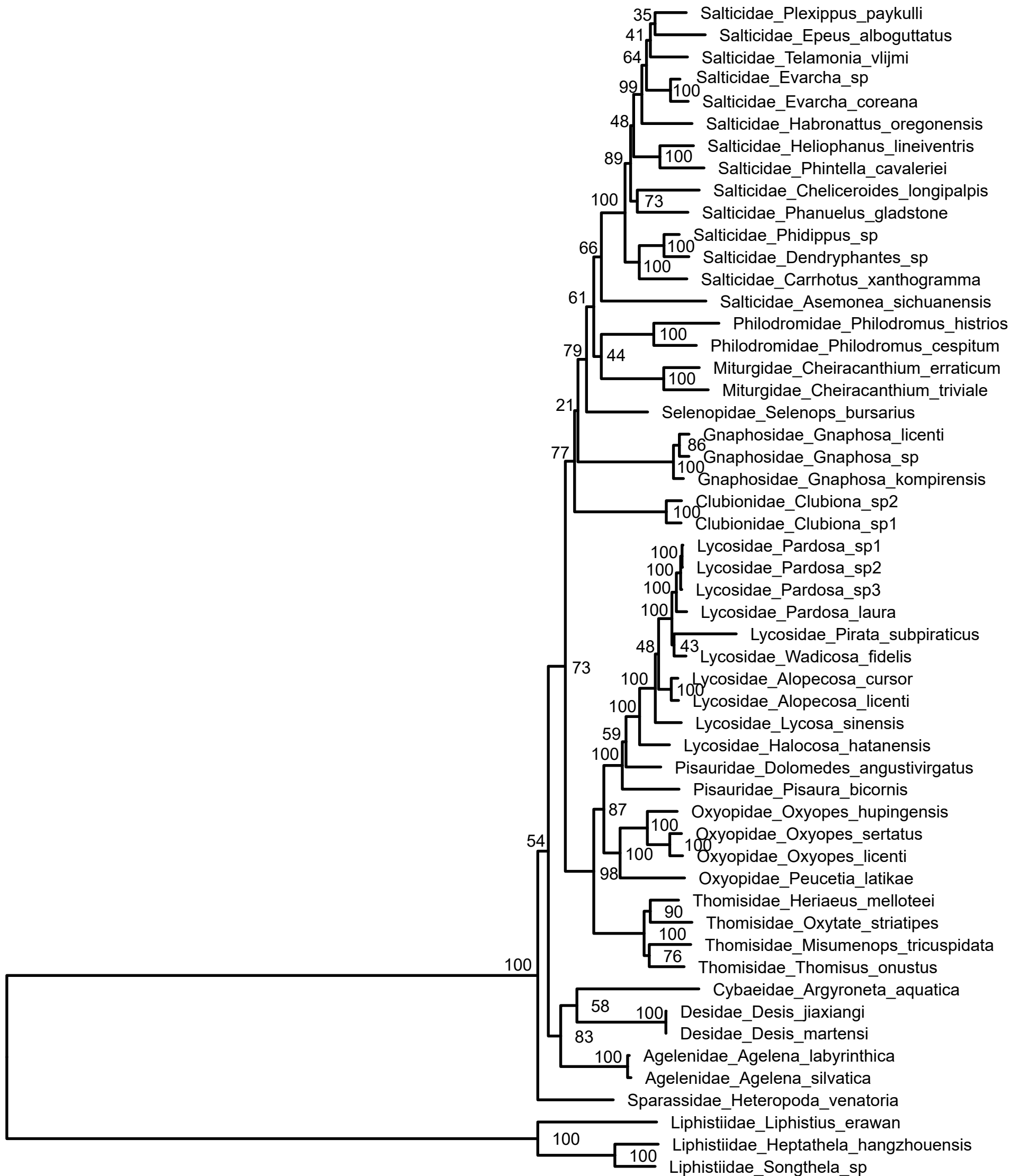

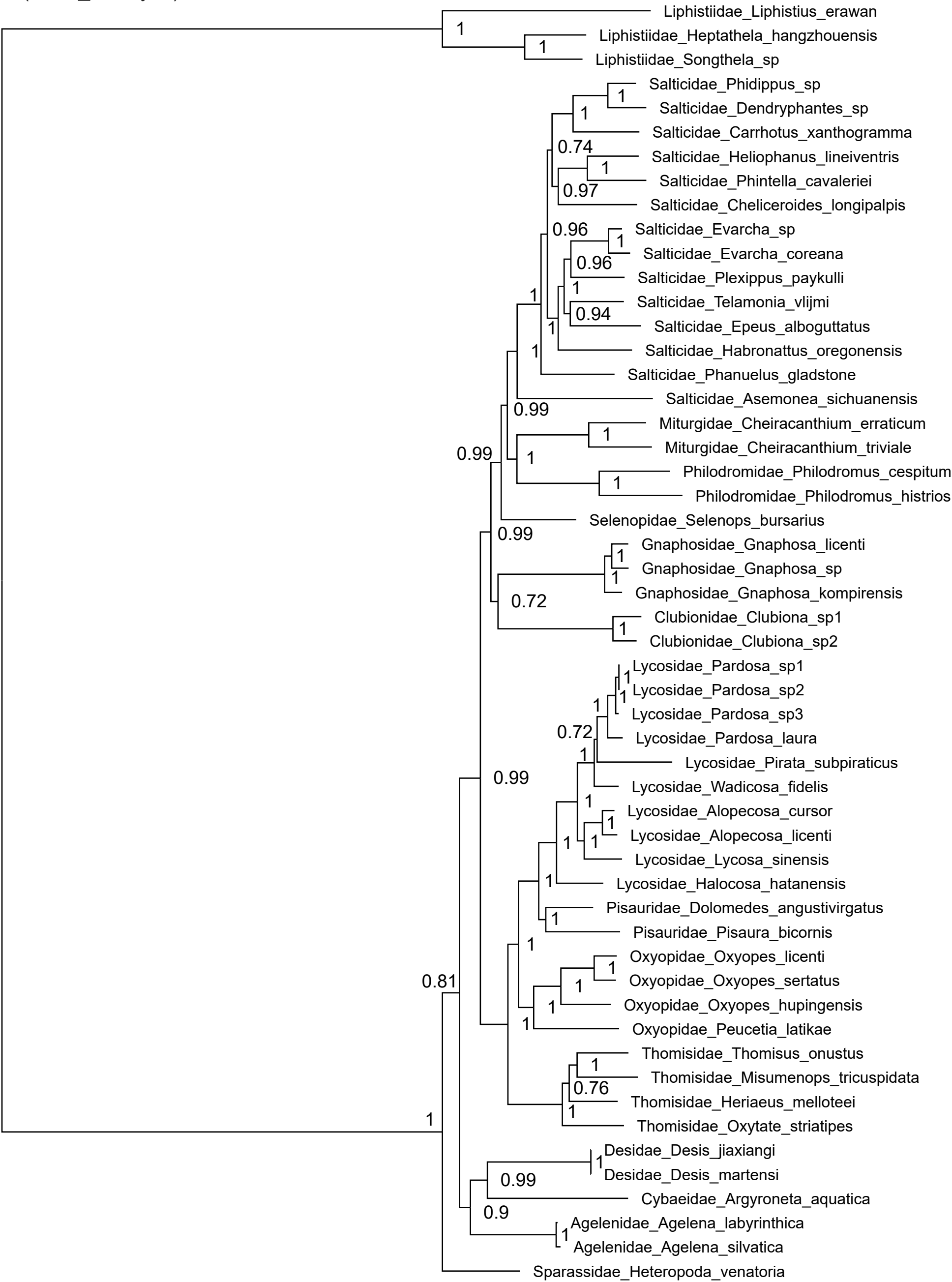

3.0

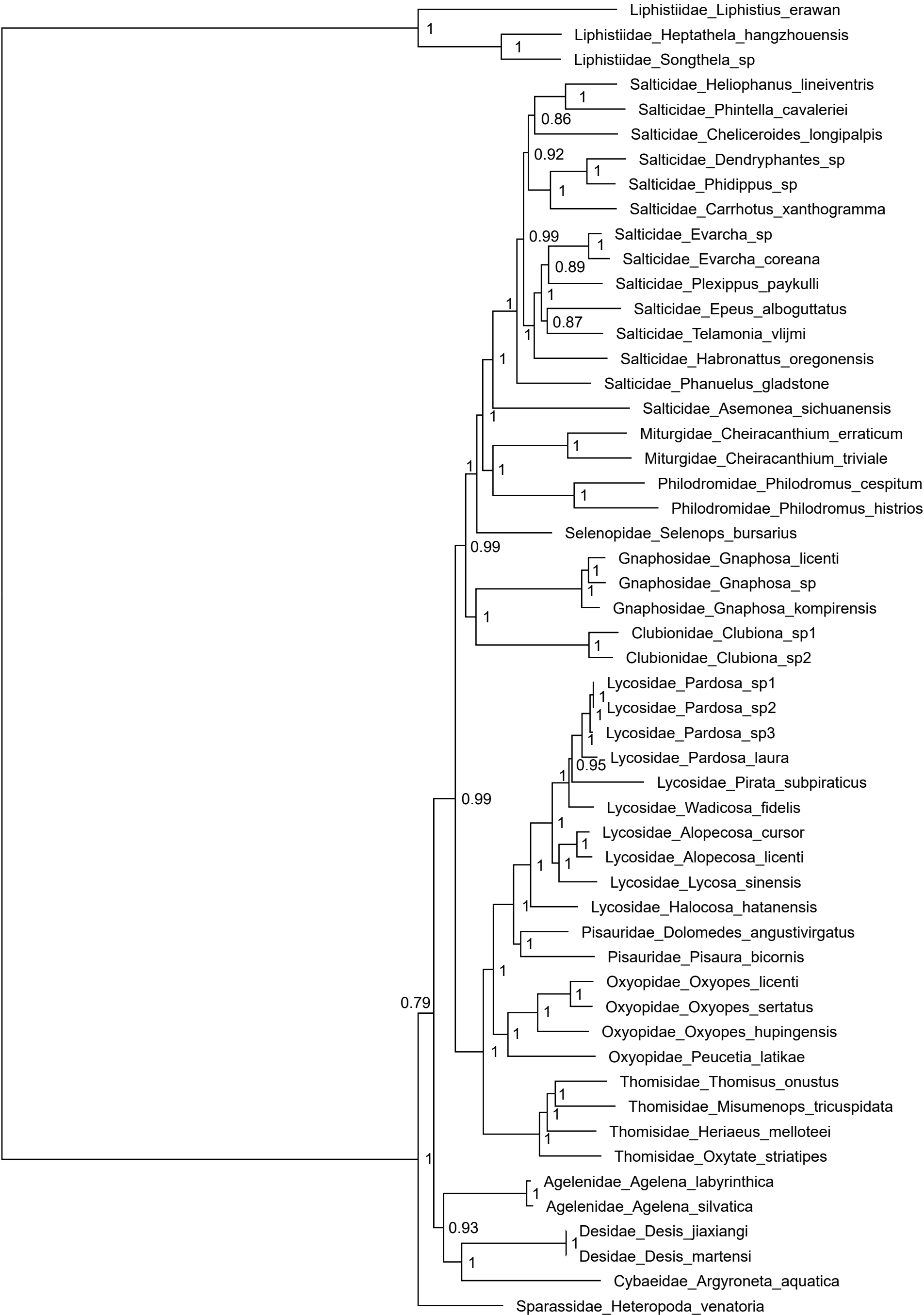

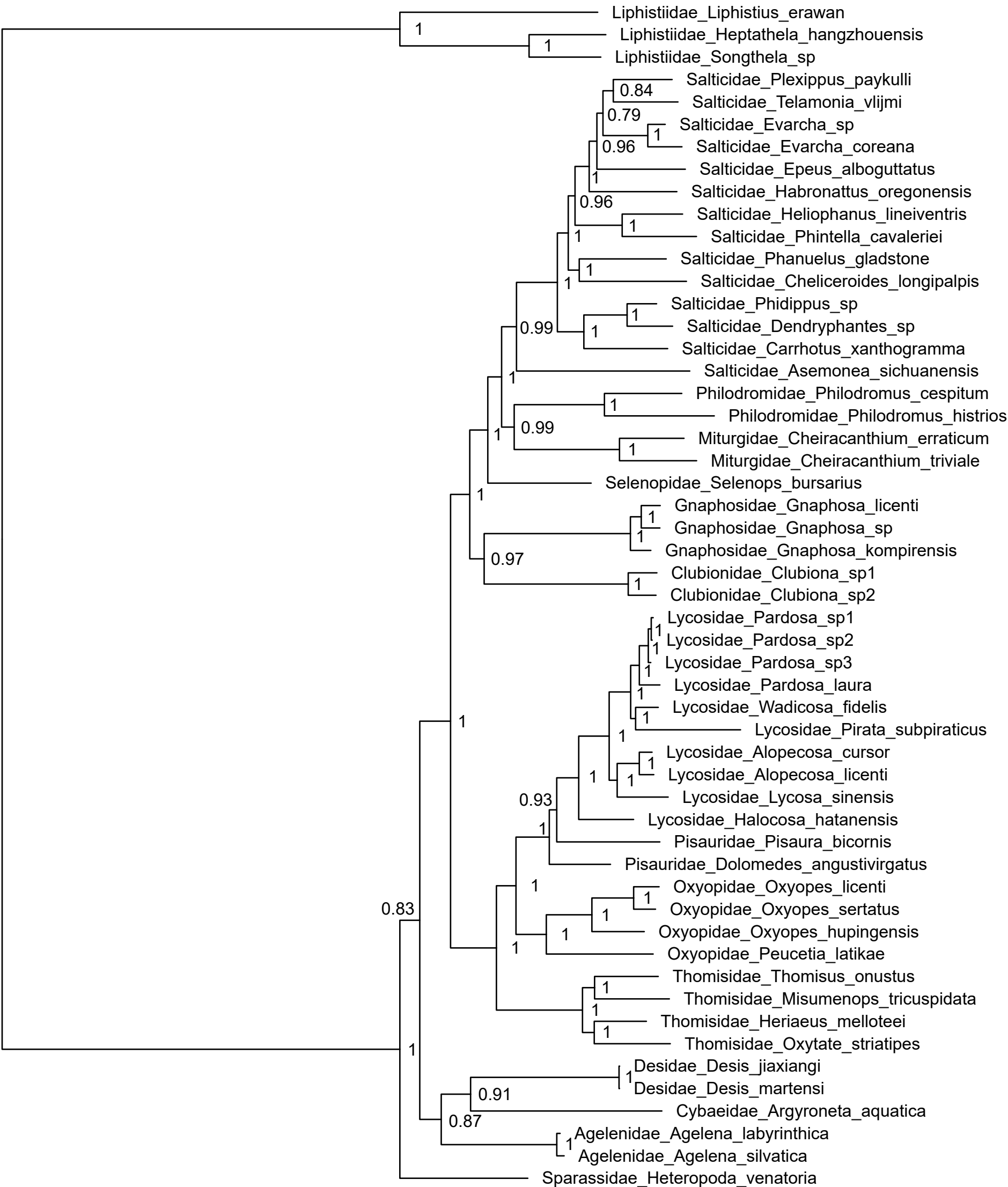

Supplement: Supplementary file 1 [file DataSheet1.ZIP › Additional files/Figure S2 Phylogenetic results.pdf]
